# *LuxS* in *Lactobacillus plantarum* SS-128 Improves the Texture of Refrigerated *Litopenaeus vannamei*: Mechanism Exploration Using a Proteomics Approach

**DOI:** 10.3389/fmicb.2022.892788

**Published:** 2022-05-31

**Authors:** Yuan Li, Yilin Qian, Xiaowei Lou, Zhiheng Hu, Yaqin Hu, Mingyong Zeng, Zunying Liu

**Affiliations:** ^1^College of Food Science and Engineering, Ocean University of China, Qingdao, China; ^2^College of Biosystems Engineering and Food Science, Zhejiang University, Hangzhou, China; ^3^Qingdao Engineering Research Center for Preservation Technology of Marine Foods, Qingdao, China; ^4^Department of Food Science and Technology, National University of Singapore, Singapore, Singapore; ^5^College of Food Science and Technology, Hainan Tropical Ocean University, Sanya, China

**Keywords:** *Lactobacillus plantarum* SS-128, *Litopenaeus vannamei*, proteomics, texture, *luxS*

## Abstract

This study illustrated the texture changes of *Shewanella baltica*-inoculated *Litopenaeus vannamei* during refrigerated storage with the exogenous addition of *Lactobacillus plantarum* SS-128. The group inoculated with SS-128 had an improved texture compared with that inoculated with the *luxS*-mutant group (Δ*luxS*). Proteomics were conducted to analyze the protein alterations in *L. vannamei* and supernatant, respectively. During storage, many texture-related proteins, including myosin heavy chain and beta-actin, were maintained due to *luxS*. Some endogenous enzymes related to the energy metabolism and hydrolysis of *L. vannamei* were downregulated. The *luxS*-induced interaction with *S. baltica* showed significant changes in the expression of some critical enzymes and pathways. The ATP-dependent zinc metalloprotease FtsH and protease subunit HslV were downregulated, and the oxidative phosphorylation and glycosaminoglycan degradation pathways in *S. baltica* were inhibited, resulting in the slow deterioration of *L. vannamei*. By exploring the mechanism underlying SS-128-led manipulation of the metabolism of spoilage bacteria, we clarified the texture maintenance mechanism of *luxS* in SS-128, providing theoretical evidence for SS-128 application in food preservation.

## Introduction

Texture is a vital aspect of aquatic quality and is one of the critical indicators for consumers to judge the product’s acceptability. Therefore, maintaining the texture after harvesting and reducing quality loss is a significant part of preserving aquatic products. Among the preservation agents used, lactic acid bacteria and their metabolites have attracted extensive attention due to their pollution-free nature and good biocontrol effect (Lili et al., 2018; Zheng et al., 2020). Studies have shown that the biocontrol lactic acid bacteria could inhibit the deterioration of aquatic products, retard the degradation of tissue proteins, maintain hardness and chewiness, and reduce the loss caused by texture deterioration, while the sensory properties of shrimp could be effectively preserved (Li et al., 2019). However, there is a lack of in-depth understanding of the texture maintenance mechanism of aquatic products, which limits the application and development of lactic acid bacteria as a biocontrol technology.

The lactic acid bacteria, generally recognized as safe (GRAS) by the FDA, has been used to prevent pathogen growth as food preservative. Using lactic acid bacteria with biocontrol function as a preservative agent has become a promising method for aquatic product preservation (Speranza et al., 2017). Biocontrol lactic acid bacteria have been proven to play an essential role in processing and storing meat and dairy products, fruits, vegetables, and aquatic products (Sidooski et al., 2019). This study focused on the preservation and antibacterial effect of biocontrol lactic acid bacteria on aquatic products. However, there is still a lack of research on the protective effects and related mechanisms of biocontrol lactic acid bacteria on the texture of aquatic products. Additionally, the effect and mechanism of biocontrol lactic acid bacteria regulating microbial metabolism on the sensory quality and texture of aquatic products are unclear.

The quorum sensing (QS) system of biocontrol lactic acid bacteria is an essential target for delaying spoilage (Hossain et al., 2021). QS is a bacterial phenomenon wherein bacteria produce and release specific signal molecules to sense changes in their concentration and coordinate group behavior (Whiteley et al., 2017). AI-2/LuxS QS is a critical QS system for the biocontrol effect of lactic acid bacteria and is mainly regulated by *luxS*. The *luxS* gene is reported to regulate the growth characteristics and bacteriostatic ability of *Lactobacillus plantarum* by nutrient competition (Qian et al., 2022). Cyclized or modified 4,5-hydroxybiphenyl2,3-pentanedione (DPD) molecules are used as signal molecules in AI-2/LuxS QS systems mediated by AI-2 signal molecules (Subramani and Jayaprakashvel, 2019). The regulation of spoilage bacteria in storage by biocontrol lactic acid bacteria QS is essential for its biocontrol function. Studies have shown that some biocontrol lactic acid bacteria can effectively decrease the deterioration of the quality and texture of shrimp during storage due to the AI-2/LuxS QS system (Saraoui et al., 2017).

*Litopenaeus vannamei* is one of the most representative aquatic products with a high demand for quality. Therefore, protecting its texture deterioration is of good economic value (Ekezie et al., 2019). Frozen storage and refrigerator storage are the most commonly used methods of refrigerator storage. However, both methods have some limitations, i.e., ice crystals cause texture damage during frozen storage, and texture deterioration is often induced by microbial reproduction and protein erosion during refrigerator storage. *Shewanella baltica* and *Pseudomonas* have been the most significant spoilage microorganisms during the storage of some aquatic products, including shrimp (Gong et al., 2020; Lou et al., 2021). Adding exogenous matters to control the destructive ability of spoilage bacteria against texture-related proteins of *L. vannamei* could maintain its structure and functional activity, effectively improving the texture retention ability. Therefore, it is necessary to establish biocontrol methods for *L. vannamei* refrigeration. A study on its ability and internal mechanism on protein alterations could be a promising approach to reduce texture deterioration.

Proteomics has become an essential molecular technique to explore the internal mechanism of protein alterations in muscle foods. Label-free and tandem mass tags (TMT) proteomics are two widely applied branches of proteomics study. Label-free proteomics is a relative quantitative proteomics technology that directly analyzes the enzymatic peptides of proteins without any stable isotope labeling. Label-free proteomics requires the samples to be treated using a simple process. The treated samples could be analyzed directly without marking. Label-free proteomics could be a desirable approach for mixed samples from multiple species to select the drug action targets and unique functional proteins (Couto et al., 2019). TMT proteomics was developed and launched by Thermo Co., Ltd. TMT reagent is an amine-labeled heavy element related to amino groups (including amino acid N-terminal and lysine side chain amino). TMT proteomics could label and analyze 10 samples (10 plex) and simultaneously compare the protein expression differences of 2–10 groups of samples, providing an accurate digital signal, high detection flux, and wide detection range. Qualitative and quantitative analyses could be conducted simultaneously, and each component’s relative expression level, molecular weight, and rich structural information can be obtained. It is especially suitable for differential protein analysis of samples with multiple processing methods or from multiple processing times (Sonnett et al., 2018).

Our laboratory has previously purified a strain of biocontrol lactic acid bacterium (*L. plantarum* SS-128, strain No. CGMCC-17003) from the intestinal tract of blackhead fish. The *luxS*-deficient strain of SS-128 strain has been constructed, and it has been proved that its AI-2/LuxS QS system could regulate the growth of lactic acid bacteria, delaying the putrefaction of *S. baltica*-inoculated *L. vannamei* (unpublished data). In this study, texture-related proteins of *L. vannamei* manipulated by the *luxS* of SS-128 were identified by label-free proteomics in tandem with TMT proteomics analysis. Furthermore, this study explored the protective ability of the landmark endogenous enzyme/microbial metabolic differential pathway of biocontrol lactic acid bacteria on texture-related differential proteins during storage of *L. vannamei* and studied the central target manipulated by *luxS* in the texture maintenance of *L. vannamei*. Therefore, this study aimed to clarify how the *luxS* manipulating SS-128 maintains the texture of *L. vannamei* during storage.

## Materials and Methods

### Materials

The shrimp (*L. vannamei*) was bought from Qidong Rd. Market, Qingdao, with a weight of 45 ± 5 g. The shrimp was transported to the laboratory on ice within 30 min.

The wild strain of *L. plantarum* SS-128 and the *luxS*-mutant strain of *L. plantarum* SS-128 (Δ*luxS*) were constructed and preserved by the Laboratory of Aquatic Products Higher Application Technology, Ocean University of China. *Shewanella baltica* is a laboratory-preserved strain previously isolated from spoiled *L. vannamei* (Zhu et al., 2015). DPD was obtained from Professor Ming Li’s lab in the College of Pharmacy, Ocean University of China. The De Man, Rogosa and Sharpe (MRS) broth, Lactobacillus medium, and agar were bought from Qingdao Haibo Biotechnology Co., Ltd., China. Na_2_HPO_4_⋅12H_2_O, NaH_2_POx⋅H_2_O, NaCl, 40% Acr-Bis, Tris–HCl, N,N,N′,N′-tetramethylethylenediamine, Tris, dithiothreitol, and other analytical pure chemical reagents were purchased from Sinopharm Group Chemical Reagent Co., Ltd.

### Bacteria Growth Assay

The *L. plantarum* SS-128 and the *luxS*-mutant strain were incubated in the MRS medium overnight at 37°C. The 1% overnight-cultured product was then incubated in MRS medium at 37°C for 24 h, with a DPD-exogenous addition group of 24 μl DPD. Next, the *S. baltica* was cultured in LB culture with a pH of 7.0 at 30°C. The absorbance was measured under 600 nm in 0, 2, 4, 6, 8, 10, 12, and 24 h. The bacterial density was expressed as OD600.

### Preparation of Inoculated Shrimp

The shrimps were peeled, decapitated, and eviscerated under ice anesthesia and then rinsed three times with sterile water on a clean bench. The shrimps were randomly divided into 5 groups. For *S. baltica*-inoculated groups, the shrimps were immersed in 10^8^ cfu/g *S. baltica* for 10 s and then taken out for further inoculation treatment. The control group (CG) was not subjected to further inoculation treatments unless the *S. baltica* inoculation. The *L. plantarum* SS-128 group was sprayed with *L. plantarum* 10^8^ cfu/g SS-128 until the shrimp surface was covered with the bacteria evenly. The *luxS*-mutant group (Δ*luxS*) was sprayed with the *luxS*-mutant strain with a concentration of 10^8^ cfu/g. The *luxS*-mutant strain and DPD-exogenous addition groups were sprayed with DPD-exogenous addition to *luxS*-mutant strain with a concentration of 10^8^ cfu/g. Besides, groups without the inoculation of any bacteria were named ordinary shrimp groups (OSG).

All samples were packed separately in plastic bags and stored in a refrigerator at 4°C.

### Texture Analysis

The texture of shrimp during storage was measured using a TA-XT2i texture analyzer (Stable Micro Systems Ltd., Godalming, United Kingdom). The second and the third sections of shrimp were placed on the platform of the texture analyzer, and a probe P5 with a 5-mm diameter flat probe. The deformation percentage was set as 30%, the test speed was set as 1 mm s^–1^, the return speed was set as 1 mm s^–1^, the trigger force was set as 5 g, and the cycle ran two times. The cohesiveness, gumminess, adhesiveness, resilience, hardness, springiness, and chewiness were measured. Each group was repeated into eight parallel groups.

### Sensory Evaluation

The sensory evaluation of shrimp was performed by a trained panel of 20 trained members from the faculty and students from the College of Food Science and Technology, who have been trained for shrimp evaluation. The panelists were asked to evaluate the color, odor, texture, appearance, and overall acceptability of the shrimp sample using a 10-point hedonic scale (0 for dislike extremely to 9 for like extremely). The sensory evaluation was performed individually under controlled conditions.

### Proteomics Analysis

**FIGURE 1:**
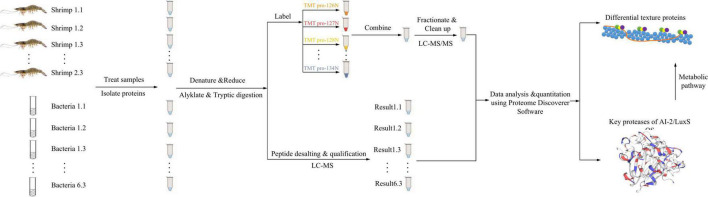
Flowchart of proteomic analysis.

The proteomics analysis of shrimp was conducted with TMT proteomics, and the analysis for surface bacteria was conducted with label-free proteomics. The summary flowchart for the proteomics analysis is shown in Figure 1, and the detailed protocol is shown below.

#### Protein Extraction

An appropriate amount of shrimp sample in the refrigerator at –80°C was transferred into a grinding tube, adding some protein lysate with 8 M urea + 1% sodium dodecyl sulfate (SDS) (containing protease inhibitor). For surface bacteria, the 0.9% (w/v) sterile saline was mixed with shrimp and shaken for 5 min in a sterile plastic bag, and then the mixed liquids were transferred into a centrifugal tube and centrifuged at 16,000 × *g* and 4°C for 30 min. The supernatant was discarded, and then some protein lysate with 8 M urea + 1% SDS (containing protease inhibitor) was added. A high-throughput tissue grinder was used to vibrate the sample three times, for 40 s each time. Then, the sample was cracked on ice for 30 min, with vortex mixing 5–10 s for every 5 min. The mixture was centrifuged at 16,000 × *g* and 4°C for 30 min, the supernatant was collected, and the protein content was measured using the bicinchoninic acid assay (Smith et al., 1985).

#### Sodium Dodecyl Sulfate-Polyacrylamide Gel Electrophoresis

The SDS-polyacrylamide gel electrophoresis protocol was modified according to Li et al. (2020c). Shrimp protein samples, which were extracted as mentioned above, were mixed with loading buffer in the ratio of 1:4 and then placed in a water bath for 10 min at 95°C. The concentration of spacer gel was 6%, and the concentration of separating gel was 10%. Each well of the gel was injected with 15 μl of the sample. The running buffer with a pH of 8.3 consisted of 28.8 g glycine, 6.0 g Tris, and 1.0 g SDS. The constant voltage power supply for cataphoresis was 100 V, running for around 2 h. The gel was then put into dyeing liquor for 1.5 h. After dyeing, the gel was rinsed three times and then placed in the detainer until the background became transparent.

#### Reductive Alkylation and Enzymatic Hydrolysis

A weight of 100 μg protein was measured and triethylammonium bicarbonate buffer (TEAB) was added to make the final concentration of 100 mM. Tris (2-carboxyethyl) phosphine was added to obtain a final concentration of 100 mM and allowed to react at 37°C for 60 min. The IAM (iodoacetamide) was added to make the final concentration of 40 mM, and the solution was held for 40 min at room temperature without light. For each tube, the precooled acetone was added in the ratio of 6:1 (acetone:sample, v/v) and then precipitated at –20°C for 4 h; centrifugation was done at 10,000 × *g* for 20 min, the precipitate was collected, and 100 μl of TEAB (100 mM) was used to treat the sample until fully dissolved. Trypsin was added according to the ratio enzyme:protein (M/M) = 1:50, and the enzymatic hydrolyses were conducted overnight at 37°C.

#### Tandem Mass Tags Labeling and First-Dimensional Separation of High pH RPLC

After performing the abovementioned procedures, the shrimp samples were processed as follows. The TMT reagent (Thermo Fisher Scientific No. a44522) was taken out at 20°C and restored to room temperature. The reagent was centrifuged, acetonitrile was added, and centrifuge was vortex mixed. A tube of TMT reagent was added for every 100 μg polypeptide and incubated at room temperature for 2 h. Then, hydroxylamine was added, and the samples were kept at room temperature for 30 min. Each group of moderately labeled products was mixed into a tube and drained using a vacuum concentrator.

The polypeptide samples were redissolved with UPLC loading buffer (2% acetonitrile with ammonia adjusted to pH 10) and separated by a reversed-phase C18 column. Column information: ACQUITY UPLC BEH C18 Column 1.7 μm, 2.1 mm × 150 mm (Waters, United States); Chromatographic instrument: Thermo Scientific Vanquish Flex Binary UHPLC system; phase A: 2% acetonitrile (ammonia adjusted to pH 10); phase B: 80% acetonitrile (adjust ammonia to pH 10); UV detection wavelength: 214 nm; flow rate: 200 μl/min; and gradient: 48 min. According to the peak type and time, 20 fractions were collected and combined into 10 fractions. After vacuum centrifugation and concentration, the fractions were dissolved in mass spectrometry loading buffer (2% acetonitrile and 0.1% formic acid) for the second-dimensional analysis.

#### Peptide Desalting and Qualification

After performing the abovementioned procedures, the surface bacteria samples were processed according to this section. After trypsin digestion, the peptide segments were dried with a vacuum pump. The digested peptides were redissolved with 0.1% trifluoroacetic acid (TFA). After desalting the peptide segments with hydrophilic and lipophilic balance and a mixed-mode cation exchanger (MCX) solid-phase extraction column, each sample was divided into two parts and dried with a vacuum concentrator. Peptide quantification was conducted using a Thermo Fisher Scientific peptide quantification kit (No. 23275).

According to the quantitative results of peptide segments, the peptide with an equal concentration of 0.25 μg/μl was dissolved in mass spectrometry loading buffer (2% acetonitrile and 0.1% formic acid) for mass spectrometry analysis.

#### Liquid Tandem Mass Spectrometry

After treating the shrimp samples and surface bacteria samples as stated above, liquid tandem mass spectrometry was performed according to the following parameters:

Data acquisition software: Thermo Xcalibur 4.0 (Thermo, United States); reversed-phase column information: C18 column (75 μm × 25 cm, Thermo, United States); chromatographic instrument: EASY-nLC 1200 (Thermo, United States); mass spectrometer: Q_Exactive HF-X (Thermo, United States); chromatographic separation time: 120 min for TMT proteomics, 90 min for label-free proteomics; phase A: 2% acetonitrile and 0.1% formic acid; phase B: 80% acetonitrile and 0.1% formic acid; and flow rate: 300 nl/min.

The EASY-nLC liquid-phase gradient for TMT proteomics was shown as follows: 0 min, 5%; 65 min, 23%; 81 min, 29%; 90 min, 38%; 92 min, 48%; 93 min, 100%; 120 min, stop. MS scanning range (m/z): 350–1,500; acquisition mode: DDA, top 15 (select the 15 with the strongest signal in the parent ion for secondary fragmentation); primary mass spectrometry resolution: 120,000; AGC target: 3e6; maximum injection time: 50 ms; fragmentation mode: HCD; secondary resolution: 45,000; AGC target: 2e5; maximum injection time: 120 ms; fixed first mass: 110 m/z; minimum AGC target: 1e4; intensity threshold: 8.3e4; and dynamic exclusion time: 30 s.

The EASY-nLC liquid-phase gradient for label-free proteomics was shown as follows: 0 min, 5%; 53 min, 23%; 65 min, 29%; 73 min, 38%; 74 min, 48%; 75 min, 100%; 90 min, stop. MS scanning range (m/z): 350–1,500; acquisition mode: DDA, top 20 (select the 20 with the strongest signal in the parent ion for secondary fragmentation); primary mass spectrometry resolution: 60,000; AGC target: 3e6; maximum injection time: 20 ms; fragmentation mode: HCD; secondary resolution: 15,000; AGC target: 1e5; maximum injection time: 50 ms; fixed first mass: 100 m/z; minimum AGC target: 8e3; intensity threshold: 1.6e5; and dynamic exclusion time: 18 s.

#### Database Search

The software version used for library search is Proteome Discoverer™ Software 2.4. When searching the database, submit the raw file to the proteome discoverer server, select the established database, and then search the database.

For TMT proteomics, the parameters were set as follows: protein database: uniprot-taxonomy-6689.unique.fasta; cys alkylation: iodoacetamide; dynamic modification: oxidation (M), acetyl (protein N-terminus), met-loss (protein N-terminus), and met-loss + acetyl (protein N-terminus); static modification: carbamidomethyl (C), TMTpro (K), and TMTpro (N-terminus); enzyme name: trypsin (Full); maximum missed cleavage sites: 2; precursor mass tolerance: 20 ppm; fragment mass tolerance: 0.02 Da; and validation based on *q*-Value. The resultant filtering parameter is peptide FDR ≤ 0.01. For label-free proteomics, the parameters were set as follows: protein database: merge-uniprot-taxonomy-1590 + 6689 + 62322.unique.fasta; cys alkylation: iodoacetamide; dynamic modification: oxidation (M), acetyl (protein N-terminus), met-loss (protein N-terminus), and met-loss + Acetyl (protein N-terminus); static modification: carbamidomethyl (C); enzyme name: trypsin (full); max. missed cleavage sites: 2; precursor mass tolerance: 10 ppm. The resultant filtering parameter is peptide FDR ≤ 0.01.

### Statistical Analyses

Except for special experiments, all experiments were performed in triplicate. The data were analyzed using analysis of variance. Pearson correlation coefficients were used for statistical correlation, and a *p*-value of <0.05 was considered statistically significant. Correlation coefficients were calculated using the program SPSS 16.0 (SPSS Inc., Chicago, IL, USA). Figures were drawn using SciDAVis software and Matlab2020a.

## Results and Discussion

### Texture and Sensory Evaluation

**FIGURE 2:**
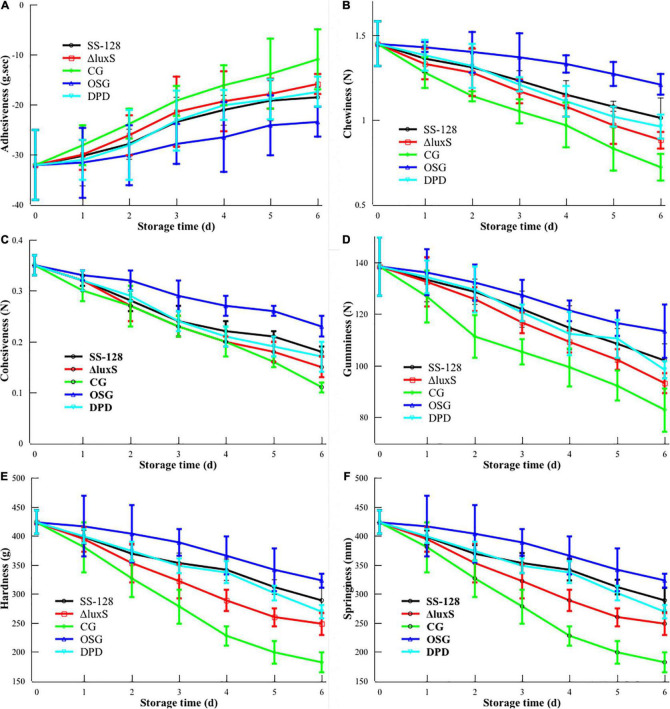
Texture changes during storage of *Litopenaeus vannamei*. **(A)** Adhesiveness; **(B)** chewiness; **(C)** cohesiveness; **(D)** gumminess; **(E)** hardness; and **(F)** springiness.

The texture is the first feeling of food in the mouth experienced by consumers. The texture analyzer tested the detailed parameters of various aspects by simulating the process of chewing in the mouth. The receptors obtained the results using probes. Freezing damage, microorganism invasion, and endogenous enzymes are common reasons that contribute to the loss of texture (Kadim et al., 2020). Storage at 4°C ruled out the interference due to ice damage. However, during the storage of *S. baltica*-inoculated *L. vannamei*, the texture could be destroyed due to spoilage microorganisms and endogenous enzymes in *L. vannamei*. Adding exogenous SS-128 also increased the factors interfering with texture during storage, and the interfering intensity may not be linear. To illustrate the texture change laws of *L. vannamei* more accurately, we analyzed the texture parameters, including adhesiveness, chewiness, cohesiveness, gumminess, hardness, and springiness each day. The results are shown in Figure 2.

Adhesiveness reflects the strength between muscle tissues (Zhou et al., 2019). The absolute value of adhesiveness decreased during storage, which corresponded with the loosening of tissue for *L. vannamei*. The evasion of spoilage microorganisms, especially after incubation with *S. baltica*, could be the primary reason for this phenomenon. As the specific spoilage microorganism of *L. vannamei*, *S. baltica* has a high proteolytic ability. Groups inoculated with *S. baltica* (SS-128, CG, DPD, and Δ*luxS*) presented a higher decrease in adhesiveness rate than the group without treatment. Similar results were shown in other indexes in the texture analysis. Our previous study has proved that the *luxS* in SS-128 could induce the AI-2/LuxS QS, and the addition of DPD in the *luxS*-mutant group could replenish the AI-2/LuxS QS to some extent (unpublished data). Li et al. (2019) found similar laws in another strain of lactic acid bacteria. The better texture of group DPD than that of the *luxS*-mutant group was evidence for the effect of the AI-2/LuxS QS system on texture maintenance.

Chewiness is used as a comprehensive index for evaluating the texture. For the four *S. baltica*-inoculated groups, the texture decline rate followed the rank: SS-128 < DPD < Δ*luxS* < CG. The previous study has proved that the AI-2/LuxS QS system could inhibit the growth of *S. baltica* and decrease the expression of protease in *S. baltica* (Li et al., 2020a; Mukherjee et al., 2020). Our previous study also obtained similar results in the coculture experiment of *S. baltica* and SS-128 (unpublished data). Those two effects delayed the damage of *S. baltica* on *L. vannamei* by reducing the amount of *S. baltica* and weakening its spoilage ability, respectively. DPD is the precursor of the AI-2 signal molecule. In the group DPD, the replenishment of DPD in *luxS*-mutant strain endows the group a similar ability in maintaining the texture of *L. vannamei* during storage (*p* > 0.05), which could be a verification of the critical role of the AI-2/LuxS QS system.

It has been reported that chewiness is correlated with hardness, springiness, and cohesiveness (He et al., 2018). The results of this study showed similar trends. Hardness characterizes the resistance of solid to invasion of external objects; the SS-128 efficiently preserves the hardness of *L. vannamei*. Springiness refers to the property that an object could recover its original size and shape after deformation, and the excellent springiness of *L. vannamei* is a vital factor in their taste preferred by consumers. The springiness of group SS-128 was well maintained. Cohesiveness is sometimes measured as the supplementary parameter of adhesiveness and gumminess. In this study, the cohesiveness change in different groups exhibited similar laws toward gumminess.

The value of cohesiveness and gumminess could reflect the tightness of protein structure (Hu et al., 2021). The gumminess of *L. vannamei* showed the significant difference from that of other typical aquatic products like hairtail due to the structure difference of their protein (Li et al., 2020b). During the storage, chewiness, hardness, springiness, cohesiveness, and gumminess decreased with time, demonstrating the spoilage of protein in *L. vannamei*.

The *luxS*-mutant group had the texture maintenance ability between group SS-128 and the control group. Although the mutant of *luxS* weakened the ability to inhibit *S. baltica* in the AI-2/LuxS QS aspect, the functional mode of biocontrol effect in SS-128 could be diversified. For instance, it could introduce bacteriocin to inhibit the growth of *S. baltica* and other microorganisms (Yi et al., 2020). So, the *luxS*-mutant group also has some protecting effect on texture during storage. Nevertheless, compared to the *luxS*-mutant group, the group SS-128 still exhibited an extraordinary effect on the texture protection (*p* < 0.05), indicating the critical role of *luxS*.

The sensory scores obtained during the storage of *L. vannamei* were shown in Supplementary Figure 1. For all of the groups, the sensory scores declined with the extension of storage, in which the group SS-128 exhibited the slowest decrease rate, corresponding to the texture results. This is a strong evidence for the feasibility of the practical application of SS-128, since some studies have found that the existence of lactic acid bacteria could affect the ordinary sensory of food. Siedler et al. (2019) reported that if used in the preservation of milk, the vinegar-like taste of acetic acid produced by lactic acid bacteria would not be favored by the customers, and the acetic environment of lactic acid bacteria could cause undesirable sensory changes in pure milk-like liquid stratification. The possible reason for the sensory maintenance in this study could be that the SS-128 did not penetrate into the shrimp meat. Another possible reason is that the shrimps have a strong buffering capacity.

### Principal Component Analysis of Proteomics Results

**FIGURE 3:**
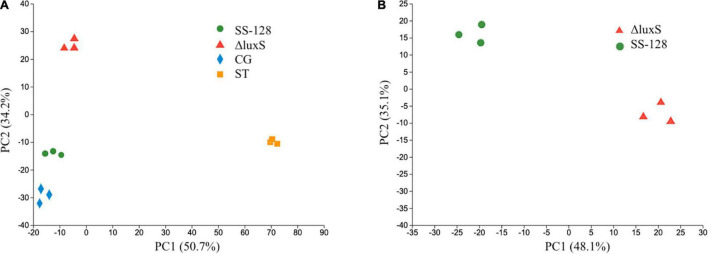
Principal component analysis scores for proteomics results of panels **(A)** *Litopenaeus vannamei* and **(B)** supernatant.

Principal component analysis (PCA) is one of the most critical dimensionality reduction methods (Lou et al., 2018). An unsupervised learning dimensionality reduction method needs eigenvalue decomposition to compress and denoise the data. Hence, it is widely used in realistic scenes for clustering analysis. In this study, the PCA analysis was conducted to evaluate the repetitiveness among parallels and the differences among groups. The PCA scores for *L. vannamei* and supernatant proteomics results are presented in Figures 3A,B, respectively. After dimension reduction analysis, there were relative coordinate points on the PCA plane. The distance between each point represented the similarity between samples. The closer distance corresponded to the higher similarity between samples.

In the PCA results of *L. vannamei*, PC1 and PC2 explained 84.9% of the total variance. The parallels of each group were clustered, indicating desirable repetitiveness. The control group, which represented the *L. vannamei* of the start point (ST), located on the positive side of PC1, whereas all the experimental groups were located on the negative side of PC1, indicating that the proteomics results of all experimental groups distinctly differed from that of the group ST. The *LuxS*-mutant group is located on the positive side of PC2, whereas the control group and SS-128 are both located on the negative part of PC2. The differences in proteomics in *L. vannamei* from group SS-128 to the *luxS*-mutant group could be considerable.

The PCA results of proteomics in the supernatant presented in Figure 3B show appreciable differences. PC1 and PC2 explained 83.2% of the total variance, and the *luxS*-mutant group and SS-128 were located in opposite parts of PC1 and PC2. Although those two groups indicated differences, the parallels in each group revealed no apparent changes.

### Proteomics Analysis of *Litopenaeus vannamei*

#### Functional Classifications of Holoproteins

**FIGURE 4:**
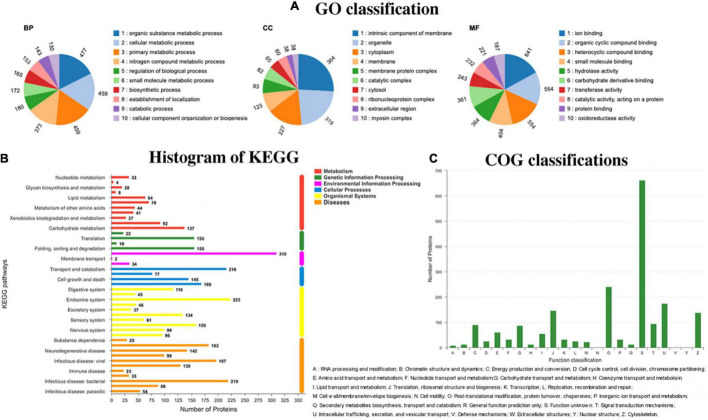
Different pathways have different functional classifications of holoproteins in *Litopenaeus vannamei*. **(A)** Go classification; **(B)** KEGG classification; and **(C)** COG classification.

All the proteins identified in the proteomics study of *L. vannamei* were classified by GO, KEGG, and COG pathways, and the results are presented in Figure 4. In the GO pathways, 1,754 proteins were identified, and the detailed GO classifications are shown in the pie charts in Figure 4A. The different colors in each pie chart represented different GO terms, the area represents the relative proportion of proteins, and the number corresponding to the color represents the number annotated to the GO term in the identified proteins. The three pie charts from left to right represented the three branches of GO, namely, biological process, cellular component, and molecular function (MF). In total, 38 kinds of proteins are classified as relating to the myosin complex. Myosin plays a vital role in cell movement and intracellular material transmission and plays a vital role in the process of muscle contraction and cell division (Scarff et al., 2020). The texture changes of *L. vannamei* could be highly related to the changes in those proteins. Except for texture-related differential proteins, there are many other differential proteins that participated in the protein activity of *L. vannamei*. Binding, catalytic activity, and cellular anatomical entity were the top 3 categories containing the largest number of differential proteins caused by the absence of the *luxS* gene.

KEGG is a practical database resource for understanding advanced functions and biological systems (such as cells, organisms, and ecosystems), providing information at the molecular level, especially genome sequencing and other high-throughput experimental technologies generated from sizable molecular data sets. In the histogram of KEGG (Figure 4B), 119 proteins were identified to relate to infectious diseases caused by the bacteria. During the storage of *L. vannamei*, the infection of diverse microorganisms and the inoculated *S. baltica* could affect those proteins.

COG is a database for the homologous classification of gene products. It is obtained by comparing many protein sequences of various organisms. As shown in Figure 4C, 138 kinds of proteins are identified to be related to the cytoskeleton. Cytoskeleton refers to the protein fiber network structure in eukaryotic cells. It plays a considerate role in maintaining cell morphology, bearing external forces, maintaining the order of internal cell structure, and participating in many important life activities. The changes in cytoskeleton-related proteins could affect the texture of *L. vannamei* during storage.

#### Differential Proteins Caused by *LuxS*

**FIGURE 5:**
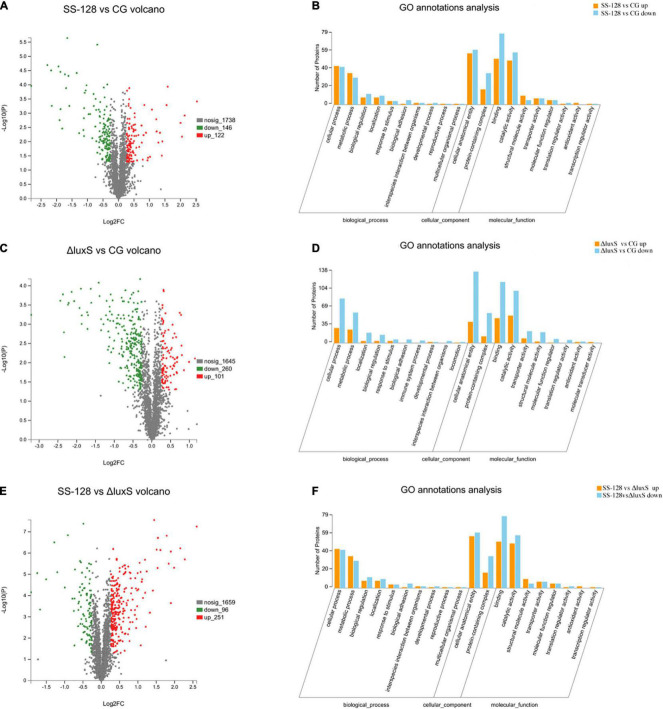
Classifications of differential proteins in *Litopenaeus vannamei*. **(A)** SS-128 vs. CG volcano; **(B)** SS-128 vs. CG GO annotation analysis; **(C)** Δ*luxS* vs. CG volcano; **(D)** Δ*luxS* vs. CG GO annotation analysis; **(E)** SS-128 vs. Δ*luxS* volcano; **(F)** SS-128 vs. Δ*luxS* GO annotation analysis.

The distribution of differential proteins in the group SS-128 and the control group on *L. vannamei* are presented in Figures 5A,B. In the volcano (Figure 5A), the abscissa represents the change value of the protein expression between samples. The ordinate was the protein expression change difference (*p*-value). The *p*-value was negatively correlated to the expression difference. The values of the abscissa and ordinate are logarithmized. Each point in the figure represents a specific protein. The point on the left is the protein with differentially downregulated expression, and the point on the right is the protein with differentially upregulated expression. In the MF of GO annotations analysis, nine kinds of differential proteins related to structural molecular activity were raised, whereas four kinds of differential proteins in this classification were reduced. Except for those proteins, some proteins classified into other classes could also have texture-maintaining functions.

For example, the degree of biological adhesion could be related to the texture (Behera et al., 2020). The top 10 differential proteins associated with the texture of *L. vannamei* are listed in Table 1A. All the quantities of those 10 proteins of *L. vannamei* were raised significantly more in the group SS-128 than in the control group, corresponding to the better texture data. The *luxS* affected the AI-2/LuxS QC system of SS-128 during the storage (Kaur et al., 2018). The SS-128 surpassed *S. baltica* in the growing competition, with the quantitative growth of SS-128, the AI-2 signals secreted more. By sensing the existence of AI-2 signals, the quantitative growth and the metabolic process of *S. baltica* could be inhibited. Although the growth and metabolism of SS-128 might use *L. vannamei* as an energy source, its damage to *L. vannamei* was far less than that from *S. baltica*, which is a recognized spoilage microorganism for aquatic products (Feng et al., 2021). The AI-2/LuxS QC system of SS-128 retarded the damage of texture proteins from microorganisms, especially from *S. baltica*. In contrast, most of the vital texture-related proteins in the control group were decomposed, resulting in the collapse of muscle structure, represented as the texture deterioration of *L. vannamei*.

**TABLE 1A:** Top 10 texture-related differential proteins in *Litopenaeus vannmei* of SS-128 vs. CG.

Protein name	Description	Change fold
Myosin heavy chain type 1	OS = Penaeus vannamei OX = 6689 GN = C7M84_024911 PE = 4 SV = 1	**9.09**
Myosin heavy chain type 1	OS = Penaeus vannamei OX = 6689 GN = C7M84_006014 PE = 4 SV = 1	**5.43**
Myosin	OS = Penaeus vannamei OX = 6689 GN = C7M84_006015 PE = 3 SV = 1	**5.08**
Actin 1	OS = Penaeus vannamei OX = 6689 GN = C7M84_001135 PE = 3 SV = 1	**5.02**
Slow muscle myosin S1 heavy chain	OS = Penaeus vannamei OX = 6689 GN = C7M84_008709 PE = 4 SV = 1	**4.72**
Lit v 3 allergen myosin light chain	OS = Penaeus vannamei OX = 6689 GN = C7M84_013542 PE = 2 SV = 1	**4.65**
Actin 1	OS = Penaeus vannamei OX = 6689 GN = C7M84_012583 PE = 3 SV = 1	**4.20**
Actin T2	OS = Penaeus vannamei OX = 6689 GN = C7M84_013443 PE = 3 SV = 1	**3.82**
Myosin light chain 2	OS = Penaeus vannamei OX = 6689 GN = C7M84_002678 PE = 4 SV = 1	**3.71**
Myosin light chain	OS = Penaeus vannamei OX = 6689 GN = C7M84_002678 PE = 4 SV = 1	**3.66**

Although the AI-2/LuxS QC system of SS-128 introduced mainly by *luxS* played a critical role in the texture maintenance of *L. vannamei*, the protection mechanisms from SS-128 could be multiplex. As shown in Figures 5C,D, numerous kinds of differential proteins were identified in *L. vannamei* between the *luxS*-mutant group and the control group, indicating that the protecting effect of *the luxS*-mutant group could be significant. These results support the phenomenon in texture analysis. Table 1B shows that in the top 10 texture-related proteins, nine kinds were raised in the *luxS*-mutant group, and eight kinds of those proteins were also identified in the differential proteins identified in SS-128 vs. CG, whereas the change folds were minor. The mutant of *luxS* weakened the texture-protecting effect of SS-128 by inhibiting the AI-2/LuxS QC system. However, the acidic environment formed by *L. plantarum* could effectively inhibit the growth of spoilage bacteria (Ruiz-Moyano et al., 2019). Since it has been reported that the optimal pH for *S. baltica* was under meta-alkalescence (Kim et al., 2017), the organic acids produced by *luxS*-mutant SS-128, including acetic acid and lactic acid could also contribute to the inhibition of spoilage microorganisms. Therefore, under an acidic environment formed by SS-128, the growth of *S. baltica* could be affected. Besides, the bacteriocin produced by *luxS*-mutant SS-128 was unfavorable for spoilage microorganisms (Bagde and Nadanathangam, 2019), because the production of some bacteriocin was shown to be unrelated to the absence of *luxS* (Wang et al., 2021).

**TABLE 1B:** Top 10 texture-related differential proteins in *Litopenaeus vannmei* of Δ*luxS* vs. CG.

Protein name	Description	Change fold
Myosin heavy chain type 1	OS = Penaeus vannamei OX = 6689 GN = C7M84_024911 PE = 4 SV = 1	**7.14**
Myosin heavy chain type 1	OS = Penaeus vannamei OX = 6689 GN = C7M84_006014 PE = 4 SV = 1	**5.00**
Slow muscle myosin S1 heavy chain	OS = Penaeus vannamei OX = 6689 GN = C7M84_008709 PE = 4 SV = 1	**4.55**
Myosin	OS = Penaeus vannamei OX = 6689 GN = C7M84_006015 PE = 3 SV = 1	**4.55**
Lit v 3 allergen myosin light chain	OS = Penaeus vannamei OX = 6689 GN = C7M84_013542 PE = 2 SV = 1	**3.85**
Myosin heavy chain type 2 (Fragment)	OS = Penaeus vannamei OX = 6689 GN = C7M84_013696 PE = 4 SV = 1	**3.70**
Actin 1	OS = Penaeus vannamei OX = 6689 GN = C7M84_012583 PE = 3 SV = 1	**3.33**
Myosin light chain 2	OS = Penaeus vannamei OX = 6689 GN = C7M84_018700 PE = 4 SV = 1	**3.23**
Myosin light chain	OS = Penaeus vannamei OX = 6689 GN = C7M84_002678 PE = 4 SV = 1	**3.13**
Tubulin alpha chain (Fragment)	OS = Penaeus vannamei OX = 6689 GN = C7M84_021811 PE = 3 SV = 1	**0.47**

When group SS-128 was compared to the *luxS*-mutant group, 347 types of proteins were identified as differentially expressed proteins (Figures 5E,F), illustrating that the *luxS* played a crucial role during the storage of *L. vannamei*. Myosin heavy chain is the basic unit of myosin and plays a vital role in ensuring the daily activity of muscle cells. Its function is to lengthen muscle fibers to produce movement and strength (Luo et al., 2019). Myosin heavy chain showed a significant increase in fold-change (Table 1C), indicating that the *luxS* in SS-128 had a substantial effect on the protection of myosin heavy chain. Beta-actin was also a structure-related protein that was protected in the group SS-128. Actin is a globular multifunctional protein family that forms microfilaments in the cytoskeleton and filaments in muscle fibers. Beta-actin coexists in most cell types as a component of the cytoskeleton and a mediator of internal cell movement, contributing to the texture performance of muscle (Wiame et al., 2018).

**TABLE 1C:** Top 10 texture-related differential proteins in *Litopenaeus vannmei* of SS-128 vs. Δ*luxS*.

Protein name	Description	Change fold
Myosin heavy chain type b	OS = Penaeus vannamei OX = 6689 GN = C7M84_013705 PE = 4 SV = 1	**3.33**
Myosin heavy chain type 1	OS = Penaeus vannamei OX = 6689 GN = C7M84_019934 PE = 4 SV = 1	**2.44**
Myosin heavy chain type 2	OS = Penaeus vannamei OX = 6689 GN = C7M84_024902 PE = 4 SV = 1	**2.33**
Skeletal muscle actin 6	OS = Penaeus vannamei OX = 6689 GN = C7M84_009276 PE = 3 SV = 1	**1.89**
Beta-actin	OS = Penaeus vannamei OX = 6689 GN = C7M84_012581 PE = 3 SV = 1	**1.64**
Beta-actin	OS = Penaeus vannamei OX = 6689 GN = C7M84_012581 PE = 3 SV = 1	**1.56**
Tubulin alpha chain (Fragment)	OS = Penaeus vannamei OX = 6689 GN = C7M84_021811 PE = 3 SV = 1	**0.27**
Actin 1	OS = Penaeus vannamei OX = 6689 GN = C7M84_001135 PE = 3 SV = 1	**0.43**
Tubulin alpha chain	OS = Penaeus vannamei OX = 6689 GN = C7M84_011240 PE = 3 SV = 1	**0.46**
Myosin heavy chain type 1	OS = Penaeus vannamei OX = 6689 GN = C7M84_008946 PE = 4 SV = 1	**0.56**

The endogenous enzymes of *L. vannamei* participate in every aspect of changes in the internal environment, and they have an important role in the regulation of spoilage. The top 10 differential endogenous enzymes in *Litopenaeus vannmei* of SS-128 vs. Δ*luxS* are shown in Table 2. The content of three types of endogenous enzymes increased with the presence of the *luxS* gene, while most of the endogenous enzymes exhibited downregulation trends. The top 2 upregulated endogenous enzymes are kinase, which participates in the phosphorylation to transfer phosphate groups from high-energy donor molecules ATP to specific target molecules, leading to changes in the state of ion channel proteins and channel gates (Steinberg and Carling, 2019). Except for the two kinases, two kinds of ATP synthase were identified as the differential endogenous enzymes, which was evidenced by the difference in energy metabolism between the *L. vannmei* in SS-128 and the *luxS-*mutant group.

**TABLE 2:** Top 10 differential endogenous enzyme in *Litopenaeus vannmei* of SS-128 vs. CG.

Protein name	Description	Change fold
Phosphoglycerate kinase	OS = Penaeus vannamei OX = 6689 GN = C7M84_020149 PE = 3 SV = 1	**3.85**
Xylulose kinase	OS = Penaeus vannamei OX = 6689 GN = C7M84_018358 PE = 3 SV = 1	**1.67**
ATP synthase subunit gamma	OS = Penaeus vannamei OX = 6689 GN = C7M84_001248 PE = 3 SV = 1	**1.66**
Putative retinol dehydrogenase 12-like	OS = Penaeus vannamei OX = 6689 GN = C7M84_016870 PE = 3 SV = 1	**0.63**
Alpha,alpha-trehalase	OS = Penaeus vannamei OX = 6689 GN = C7M84_011816 PE = 3 SV = 1	**0.54**
Peptidyl-prolyl cis-trans isomerase	OS = Penaeus vannamei OX = 6689 GN = C7M84_023821 PE = 4 SV = 1	**0.52**
Epoxide hydrolase	OS = Penaeus vannamei OX = 6689 GN = C7M84_013199 PE = 3 SV = 1	**0.51**
3-hydroxybutyrate dehydrogenase	OS = Penaeus vannamei OX = 6689 GN = C7M84_009554 PE = 3 SV = 1	**0.48**
ATP synthase subunit gamma	OS = Penaeus vannamei OX = 6689 GN = ATP3 PE = 2 SV = 1	**0.28**
Alpha-L-fucosidase	OS = Penaeus vannamei OX = 6689 GN = C7M84_010472 PE = 3 SV = 1	**0.23**

Of the 7 kinds of downregulated endogenous enzymes, three are hydrolase (alpha-L-fucosidase, epoxide hydrolase, and alpha, alpha-trehalase), two are dehydrogenase (putative retinol dehydrogenase 12-like and 3-hydroxybutyrate dehydrogenase), and one is a hydrolase. The spoilage of muscle food could be highly related to the effect of the endogenous hydrolase, and the downregulation of the hydrolase indicated the slower structure deterioration of *L. vannmei* in group SS-128. The downregulation of dehydrogenase could be an indicator of low-level redox reactions for *Litopenaeus vannmei* in group SS-128 (Kuk et al., 2019). The results of differential endogenous enzymes showed that the existence of *luxS* in SS-128 could maintain the texture and quality of *Litopenaeus vannmei* by inhibiting the hydrolysis and energy metabolism.

### Proteomics Analysis of Supernatant

#### Overview of Differential Proteins Classification

The metabolism of endogenous enzymes and enzymes in spoilage microorganisms are considered the two primary reasons related to the texture change of muscle foods (Dehghani et al., 2018). Thus, the differences in crucial enzymes’ expression between the group SS-128 and the *luxS*-mutant group could be related to the texture. This section collected the supernatant fluid of *L. vannamei* at the endpoint of storage in the group SS-128 for label-free proteomics analysis. According to the origination, the supernatant fluid mainly contained four types of proteins: proteins from *L. plantarum* (wild strain or *luxS*-mutant strain), proteins from spoilage bacteria (in this study, only analyzed those from *S. baltica*), endogenous enzymes from *L. vannamei*, and other soluble proteins from *L. vannamei*.

**FIGURE 6:**
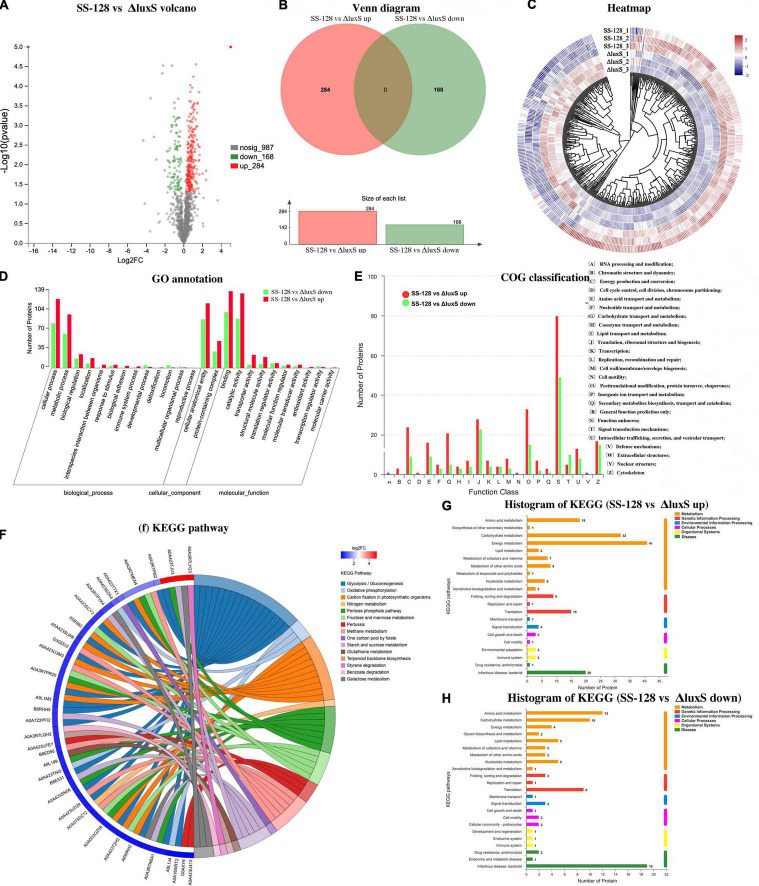
Classifications of differential proteins in the supernatant (SS-128 vs. Δ*luxS*). **(A)** Volcano; **(B)** venn diagram; **(C)** heatmap; **(D)** Go annotation; **(E)** COG classification; **(F)** KEGG pathways; **(G)** histogram of KEGG (SS-128 vs. Δ*luxS* up); and **(H)** histogram of KEGG (SS-128 vs. Δ*luxS* down).

Figure 6 provides an excellent illustration of all the identified differential proteins. Figures 6A,B shows that 284 kinds of upregulated proteins and 168 kinds of downregulated proteins were identified. Figure 6C shows the heatmap of differential proteins. In the tree view of sample clustering, the closer branches of the two samples were positively related to the closer expression patterns. The GO annotation summary in Figure 6D presents that 89 kinds of differential proteins participating in the catalytic activity were upregulated, whereas 135 were downregulated. The expression changes of those proteins might influence the deterioration degree of texture-related proteins in *L. vannamei*. COG classification in Figure 6E indicates that four kinds of proteins regarding coenzyme transportation and metabolism were upregulated and three kinds were downregulated; 15 kinds of differential proteins in signal transduction were identified. KEGG enrichment chord in Figure 6F and Table 3 presents the corresponding relationship between the target protein set and the annotation and enrichment of the KEGG pathway, and the top 50 target proteins with the most annotated pathways and the pathways with the enrichment significant *p*-value of the pathways containing these target proteins in the enrichment results were selected for display. The pentose phosphate and oxidative phosphorylation pathways in some proteins are upregulated. The histograms of KEGG are displayed in Figures 6G,H.

**TABLE 3:** Differential protein enrichment chord in supernatant (SS-128 vs. Δ*luxS*).

Accession	KEGG Pathway/GO	logFC
A0A423TJV3	Glycolysis/Gluconeogenesis, Pentose phosphate pathway, Starch and sucrose metabolism, Galactose metabolism	5
A0A3R7LYG3	Styrene degradation	5
A0A3R7PI62	Glycolysis/Gluconeogenesis	1.544
A0A3R7MEN4	Glycolysis/Gluconeogenesis, Carbon fixation in photosynthetic organisms, Pentose phosphate pathway, Pentose phosphate pathway, Fructose and	1.487
A0A423T7 × 1	One carbon pool by folate	1.311
A0A423SZA4	Oxidative phosphorylation	1.21
A0A3R7PVK4	Starch and sucrose metabolism, Galactose metabolism	0.902
A0A423SCY3	Glycolysis/Gluconeogenesis, Carbon fixation in photosynthetic organisms	0.819
K0E682	Glycolysis/Gluconeogenesis, Carbon fixation in photosynthetic organisms, Fructose and mannose metabolism	0.794
A0A423SLW8	Carbon fixation in photosynthetic organisms	0.779
G3GDU2	Glycolysis/Gluconeogenesis, Methane metabolism	0.772
A0A423U3M3	Carbon fixation in photosynthetic organisms	0.768
A0A3R7PR20	Glycolysis/Gluconeogenesis, Carbon fixation in photosynthetic organisms, Pentose phosphate pathway, Fructose and mannose metabolism, Methane mannose metabolism, Methane metabolism	0.747
A9L1M3	Glycolysis/Gluconeogenesis, Carbon fixation in photosynthetic organisms	0.736
B6RHH5	Glycolysis/Gluconeogenesis	0.736
A0A7Z2PFI2	Glycolysis/Gluconeogenesis, Starch and sucrose metabolism	0.733
A0A3R7LQH2	Terpenoid backbone biosynthesis, Benzoate degradation	0.717
A0A423U7E7	Pertussis	0.712
B8ED95	Methane metabolism	0.661
A9L199	Pentose phosphate pathway, Glutathione metabolism	0.624
A0A423TNI2	Oxidative phosphorylation	0.602
B8E531	Glycolysis/Gluconeogenesis	0.597
A0A423SN06	Glycolysis/Gluconeogenesis, Pentose phosphate pathway, Starch and sucrose metabolism	0.59
A0A423U2Q9	Glycolysis/Gluconeogenesis, Methane metabolism	0.571
A0A075DZ12	Nitrogen metabolism	0.569
A0A0D3QZ08	Glycolysis/Gluconeogenesis, Carbon fixation in photosynthetic organisms, Pentose phosphate pathway, Fructose and mannose metabolism, Methane metabolism	0.534
A0A423T2H2	Carbon fixation in photosynthetic organisms	0.507
A0A3R7N0A1	Glycolysis/Gluconeogenesis, Pentose phosphate pathway, Starch and sucrose metabolism, Galactose metabolism	0.482
A6WRH2	Glycolysis/Gluconeogenesis, Carbon fixation in photosynthetic organisms, Fructose and mannose metabolism	0.482
A9L1J4	Oxidative phosphorylation	0.382
A0A165IRT2	Glycolysis/Gluconeogenesis	0.373
G0AXY6	Pertussis	0.319
A0A423U413	Pertussis	0.303

The overview of differential proteins proved that the enzyme expression caused by *luxS* had significant differences, which could directly influence the texture of *L. vannamei* by enzymatic kinetics.

#### Differential Proteins Analysis

**FIGURE 7:**
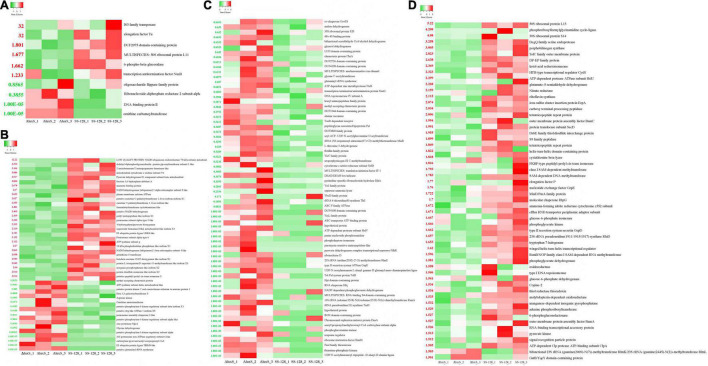
Heatmap of differential proteins in supernatant. **(A)** Differential proteins between SS-128 and Δ*luxS*; **(B)** differential enzymatic proteins (change fold > 2 or < 0.5) in *Litopenaeus vannamei*; **(C)** downregulated (change fold < 0.67) differential proteins in *Shewanella baltica*; and **(D)** upregulated (change fold > 2) differential proteins in *Shewanella baltica*.

For the differential proteins between SS-128 and its *luxS*-mutant strain, six were upregulated, and four were downregulated (Figure 7A). Those changes resulted from the *luxS* and the competitive inhibition with another microorganism, which was mainly *S. baltica* in this study. IS3 family transposase and elongation factor TU were significantly more highly expressed in SS-128 than in the *luxS*-mutant strain, whereas the DNA-binding protein II and ornithine carbamoyltransferase were concentrated in the *luxS*-mutant strain. Furthermore, it was reported that the expression of DNA-binding protein was related to the AI-2/LuxS QS system (Niazy, 2021). The 6-phospho-beta-glucosidase was upregulated in SS-128, which participates in the energy metabolism (Acin-Albiac et al., 2021).

In the supernatant, the differential proteins identified in *L. vannamei* could be divided into endogenous enzymes and other soluble proteins from shrimp muscle. The enzymes could affect the spoilage degree of *L. vannamei*, and the differential expressed enzymes in *L. vannamei* are listed in Figure 7B. Compared to the *luxS*-mutant strain, some proteases (proteasome assembly chaperone 2-like, zinc protease Mpc1, and 26S proteasome non-ATPase regulatory subunit 6-like) were downregulated in group SS-128, which retarded the degradation rate of proteins and maintained the texture of *L. vannamei*. However, some energy-consumption-related enzymes were upregulated in the *luxS*-mutant strain: NADH dehydrogenase is an enzyme located in the inner membrane of mitochondria that catalyzes the transfer of electrons from NADH to coenzyme Q (Ishikawa et al., 2020). This enzyme is an “entry enzyme” for oxidative phosphorylation in mitochondria (Guo et al., 2018). ATP synthase subunit g catalyzes the synthesis of the energy substance ATP in cells. These enzyme activities indicate the higher metabolic rate of *L. vannamei* in this group. That might lead to a faster spoilage rate, worsening the texture. Putative protein kinase C and casein kinase substrate in neurons protein 1, arginine kinase Lit v 2, putative phosphorylase b kinase regulatory subunit beta isoform X1, and putative protein kinase C and casein kinase substrate in neurons protein 1 were four downregulated kinases in the group SS-128. Protein kinase catalyzes the transfer of ATP phosphate group to substrate protein amino acid residues, called protein phosphorylation, and plays a vital role in cell signal transduction. Protein phosphorylation is the most basic, universal, and essential mechanism regulating and controlling protein activity and function (Batista et al., 2020). After phosphorylation, the protein has an electric charge, which changes the structure and further causes the change of protein activity. The lower content of those kinases in group SS-128 was consistent with the slower spoilage rate in this group.

Although endogenous enzymes in *L. vannamei* play essential roles in the texture changes during storage, the damage from exogenous spoilage microorganisms could be fiercer. As previously illustrated, the AI-2/LuxS QC system of SS-128 contributed to the texture maintenance through competitive inhibition toward *S. baltica*, and the proteins affected by *luxS* in *S. baltica* are listed in Figures 7C,D.

In the group SS-128, the content of thiamine-phosphate kinase was significantly decreased. Phosphokinase is a class of the enzyme that catalyzes the transfer of phosphate groups from ATP to other compounds. The purpose of phosphorylation is to “activate” or “enable” substrate molecules and increase their energy to participate in the reaction of subsequent negative changes in free energy. Protein kinases act on specific proteins and alter their activities (Leopold et al., 2018). The decreasing content of thiamine-phosphate kinase corresponded to the slower growth rate and metabolic strength of *S. baltica* (Rodionov et al., 2017). Phosphorylation could activate or inactivate an enzyme. The AI-2/LuxS QC system of SS-128 could affect the critical phosphorylated proteins and phosphorylation sites in *S. baltica*, control its metabolic pathways, and change the ability to deteriorate the shrimp muscle.

The ATP-dependent protease subunit HslV and zinc metalloprotease FtsH were two major downregulated differential proteins. The decreased expression of protease leads to more negligible protein hydrolysis and destruction in *L. vannamei*. *Shewanella baltica* preferentially produces elastase, collagenase, trypsin, and other proteases with vigorous enzyme activity. Those enzymes promoted protein decomposition in muscle, caused mucous membranes, and increased TVB-N release to accelerate the corruption and deterioration of *L. vannamei* (Odeyemi et al., 2020). Rather than directly affecting the secretion of protease, the AI-2/LuxS QC system of SS-128 was more likely to adjust its content by regulating the growth and metabolism of *S. baltica*. In the essential proteins that might be directly regulated, kinases play several roles in cell signal transduction and complex life activities (Jiao et al., 2018), and many phosphorylases were identified as differential proteins.

### Comprehensively Analysis of Proteomics Analysis

**FIGURE 8:**
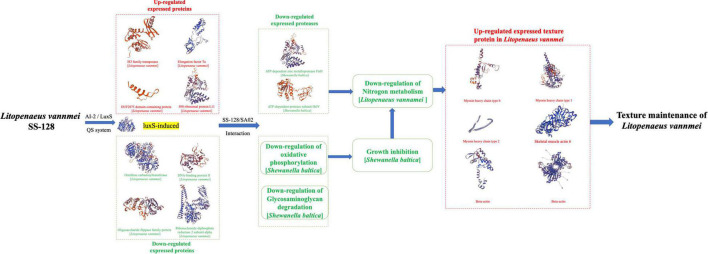
The mechanism of how SS-128 affects the texture of *Litopenaeus vannamei* in the presence of *luxS*.

Comprehensively analyzing the proteomics results by both TMT and label-free methods, the mechanism of how SS-128 affects the texture of *L. vannamei* in the presence of *luxS* is illustrated in Figure 8. The *luxS* participated in the expression of various proteins. Four proteins in SS-128 (IS3 family transposase, Elongation factor Tu, DUF2075 domain-containing protein, 50S ribosomal protein L11) were identified as the upregulated proteins, and another four proteins (ornithine carbamoyltransferase, DNA-binding protein II, oligosaccharide flippase family protein, and ribonucleoside-diphosphate reductase two subunit alpha) were identified as downregulated proteins. The *luxS* induced differences in the expression of *S. baltica* mainly in two ways: (1) Some expression of essential proteases in *S. baltica* (e.g., ATP-dependent zinc metalloprotease FtsH and ATP-dependent protease subunit HslV) were downregulated, decreasing the deterioration degree of protein in *L. vannamei*, manifested as the retard of nitrogen metabolism. (2) The critical metabolic pathways related to the cell breath and energy transfer (e.g., oxidative phosphorylation and glycosaminoglycan degradation) were downregulated, inhibiting the growth and multiplying of *S. baltica* and decreasing total spoilage bacteria amount leading to the downregulation of nitrogen metabolism. The less deterioration of nitrogen-based molecules resulted in maintaining the most vital texture protein in *L. vannamei*, providing it with a more desirable texture.

In addition to the AI-2/LuxS QC system, the SS-128 could also maintain the texture of *L. vannamei* other approaches, including the secretion of bacteriocin and inhibiting microbial activity by acidification. All those approaches together made SS-128 an intelligent agent to be used to preserve aquatic products. Furthermore, our study showed that the presence of *luxS* could already have a significant preservation effect of texture on *L. vannamei*.

## Conclusion

The *L. plantarum* SS-128 exhibited an extraordinary effect on the texture maintenance of *L. vannamei* during storage, and this study proved the critical role of *luxS* in the preservation process. The metabolic pathways induced by *luxS*, including the AI-2/LuxS QC system, could affect the multiplication mode and expression level of *S. baltica*. The absolute value of *S. baltica* decreased the invasion degree of *L. vannamei*, whereas the downregulation of some essential proteases in *S. baltica* decreased its damage to the muscle. Most of the texture-related proteins were preserved in the absence of *L. vannamei*. This study identified two proteins as the differential proteases of *S. baltica* induced by the *luxS* in SS-128, and two pathways were selected as the critical metabolic pathways affecting the growth of *S. baltica* during storage. Three myosin heavy chains, two beta-actin, and skeleton muscle actin in *L. vannamei* were identified as the significant texture-related proteins maintained by the *luxS*. Maintaining those proteins was an important reason for the better texture maintenance during the preservation. We figured out the texture maintenance mechanism of *luxS* in SS-128. This study clarified the theoretical basis of the biocontrol effect of *L. vannamei* in aquatic products, providing a target for proteins and genes for further preservative application.

## Data Availability Statement

The original contributions presented in the study are included in the article/Supplementary Material, further inquiries can be directed to the corresponding author/s.

## Author Contributions

YL did the experiment and wrote the manuscript. YQ did the experiment. XL carried out the data analysis. ZH did the experiment. YH wrote the manuscript. MZ and ZL supported the funding. All authors contributed to the article and approved the submitted version.

## Conflict of Interest

The authors declare that the research was conducted in the absence of any commercial or financial relationships that could be construed as a potential conflict of interest.

## Publisher’s Note

All claims expressed in this article are solely those of the authors and do not necessarily represent those of their affiliated organizations, or those of the publisher, the editors and the reviewers. Any product that may be evaluated in this article, or claim that may be made by its manufacturer, is not guaranteed or endorsed by the publisher.

## Supplementary Material

Supplementary Figure 1 Sensory evaluation during storage of *Litopenaeus vannamei*.
