# Supplementary figures and images for: LuxS in Lactobacillus plantarum SS-128 Improves the Texture of Refrigerated Litopenaeus vannamei: Mechanism Exploration Using a Proteomics Approach
Source: Front Microbiol. 2022 May 31;13:892788. doi: 10.3389/fmicb.2022.892788 (PMC9195002; doi:10.3389/fmicb.2022.892788)

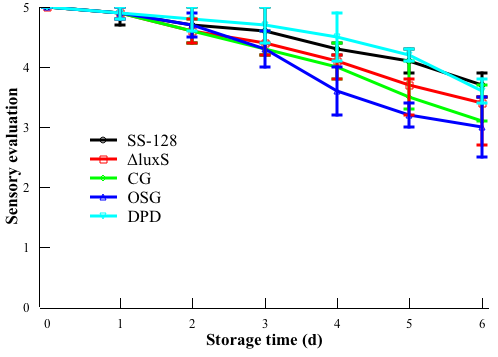

Supplement: Supplementary Figure 1 — Sensory evaluation during storage of Litopenaeus vannamei. [file Image_1.TIFF]
